# Inter-dependency between surface morphology and sensitive low RH detection: exploration of an intricate mechanism to extend the lower detection limit

**DOI:** 10.1039/d0na00047g

**Published:** 2020-05-14

**Authors:** Kusum Sharma, Noor Alam, S. S. Islam

**Affiliations:** Centre for Nanoscience and Nanotechnology, Jamia Millia Islamia (A Central University) New Delhi-110025 India sislam@jmi.ac.in +91(11)26987153

## Abstract

The water vapor molecular dynamics inside a pore structure control both molecular adsorption and desorption processes and the limit of minimum detection (LOD). Pore morphology design, and a higher concentration of electrolyte-driven anions, in accordance with the kinetics of water vapor molecules, is reported here, as the ultimate answer to extremely low relative humidity (RH) detection. In this report, a series of samples were prepared by anodization in different voltage windows, related to specific electrolyte solutions. The sensing attributes comprised: (i) a LOD of ∼3 RH%, (ii) excellent response time (6 s) and recovery time (54 s), and (iii) a hysteresis loss of ∼0.36%, with sustained stability over the period of one year; all these occurring in a sample with a pore diameter ∼5 nm ±3 nm. Interestingly, the LOD extend towards a lower RH% with a decrease in pore diameter; and a suitable explanation is given for the entire range of humidity level, in terms of the molecular mean free path, loss of kinetic energy due to scattering inside the pores, and subsequent overall loss of Brownian energy of the molecules. It is inferred from the sensing response characteristics that pore morphology and lower detection limit are interrelated; therefore, a further extension in LOD from extremely low RH% to trace levels, needs careful engineering of the pore morphology and parameters related to molecular kinetics.

## Abstract 2

Water vapor molecular dynamics, pore size, and anion concentration within the pores are interdependent and together affect the lower detection limit (LOD) and sensitivity of a humidity sensor.

## Graphical abstract

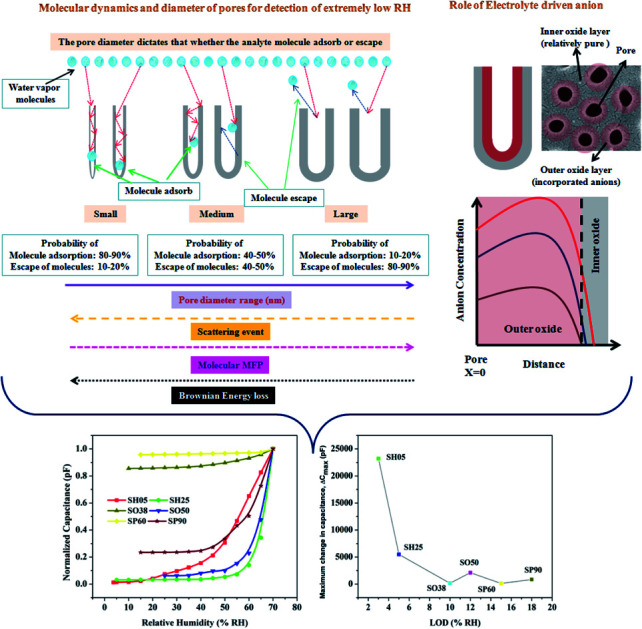

## 1. Introduction

Humidity measurement is playing an ever-increasing role in industrial, laboratory and process control. Over the years, a wide variety of humidity sensing devices have been developed. Most commercially available sensors are based on polymers or ceramics.^1–8^ Among ceramics, alumina is the most extensively employed material for humidity sensing applications, owing to its high thermal and chemical stability, hygroscopic nature and mechanical strength.^9^Table 1 compares the performance of commercially available and recently reported humidity sensors. Several techniques are available to make porous alumina, such as chemical vapor deposition (CVD), sputtering and sol–gel processes.^10^ However, all of these techniques have a common issue: *i.e.* the lack of control over the developed morphology. The electrochemical anodization method, on the other hand, has a niche advantage in developing a tailor-made porous anodic alumina (PAA) nanostructure where the pore diameter, pore depth and pore widening may be controlled easily.^9^ In terms of structural aspects, the incorporation of anions and the design of electrodes work together synergistically to govern the sensing mechanism in a nano-structured PAA.

**Table 1:** Performance comparison of commercially available and recently reported humidity sensors

Company name/model number	Active material	Detection range	Response time (s)	Recovery time (s)	Reference
—	WS_2_	20–90 RH%	5	6	11
—	GO foam	00–86 RH%	∼0.089	∼0.189	12
—	RGO/PU	10–70 RH%	3.5	7	13
GE panametrics/M series	Aluminium oxide	140–166 °F	<5	—	14
COSAXentaur/ESS-SCVP	Aluminium oxide	0.014–23 700 ppm_v_	450	—	15
COSAXentaur/Type 81/82	Polymer	0–100 RH%	—	—	16
SHAW/SADP	Aluminium oxide	0–1000 ppm_V_	<20	<120	17
—	Porous anodic alumina	15–80 RH%	—	—	18
—	Porous anodic alumina	3–90 RH%	6	54	Present work

The measurement of high relative humidity (RH) is not an issue at all, but it certainly is for low RH. Lots of R&D work is underway globally on precursor materials, surface morphology and engineering aspects to check for improvements in the lower detection limit (LOD), sensitivity, long term drift, and hysteresis. Investigations on PAA-based humidity sensors are plentiful and have been reported elsewhere.^19–25^ Y. Kim *et al.* investigated the effect of electrode design on humidity sensing,^22^ L. Yao *et al.*^23^ explained the effect of isotropic etching of pores on sensing behavior, and Nahar^24^ studied the performance degradation of PAA sensors upon exposure to high humidity. S. W. Cheng *et al.*^18^ reported sensitivity enhancement by application of a magnetic field. Presently, none of the reported humidity sensors shows any significant change in capacitance/impedance below 30 RH%.^19–25^ Even when sensing with high sensitivity, the fastest response–recovery, high resolution and stability also arouse great concern for both high- and low-level RH. Again, the quantitative sensing response is a matter of concern, irrespective of sensing level. Therefore, a complete analysis is required to bridge the gap to appreciate the interplay of pore morphology and sensor performance.

In our previous research article,^9^ the PAA nanostructure was optimized for humidity sensing applications; and the impacts of various surface pre-processing parameters were discussed in detail. In continuation of that, the present investigation reports a study of the surface morphology dependent humidity sensing response of PAA. For this, a series of samples were prepared by anodization in different voltage windows, related to specific electrolyte solutions. The underlying principles of PAA fabrication, morphology design and internal molecular dynamics, control the adsorption and desorption processes, and the sensing attributes of the sensor.

## 2. Material synthesis and sensor preparation

### 2.1. Fabrication of PAA

Aluminium sheets of high purity (99.997%, Alfa Aesar) were annealed and electropolished in a 1 : 4 ratio of perchloric acid, and ethanol solution. The process of electropolishing and its optimization were explained earlier in our previous research article.^9^ The electropolishing was carried out in galvanostatic mode at 500 mA for 80 seconds at 3 °C to create a reproducible surface finish. The aluminium sheets were then ultrasonicated in acetone in order to remove loosely bound impurities, if any, present on the surface of the Al sheet.

#### 2.1.1. Anodization

The pore diameter of PAA depends directly on the anodizing voltage. Therefore, samples with different surface morphologies were obtained by anodizing at different anodic polarizations. However, anodization voltage is electrolyte specific; there exists a narrow window of voltage in which the generation of a uniform, symmetrical and self-assembled porous structure with a specific range of pore diameters can occur. Table 2 summarizes the anodization voltage window and the related pore diameter range for the electrolytes used in the present investigation.

**Table 2:** Voltage range that can be applied safely for a given electrolyte along with the obtainable pore diameter range

S. no.	Electrolyte	Voltage range (V)	Pore diameter range (nm)
1	Sulfuric acid	5–25	2–25
2	Oxalic acid	38–50	33–90
3	Phosphoric acid	60–195	67–200

It can be observed from Table 2 that the voltage range is lowest for sulfuric acid, medium for oxalic acid and maximum for phosphoric acid solution among the reported electrolytes in the present investigation. In each electrolyte solution, two samples were anodized (as detailed in Table 3) and thus a total of six samples with six different morphologies were prepared and investigated for their humidity sensing response. Samples SH05 and SH25 were anodized at 5 V and 25 V in sulfuric acid solution; samples SO38 and SO50 were anodized at 38 V and 50 V in oxalic acid solution and finally samples SP60 and SP90 were anodized at 60 V and 90 V in phosphoric acid solution. All the samples were anodized for 3 h at temperatures below 5 °C.

**Table 3:** The anodizing parameters used for the fabrication of different morphologies of PAA

Anodization			Post anodization treatment	
Electrolyte	Sample name	Anodization voltage (V)	Etching temperature (°C)	Etching time (s)
0.3 M H_2_SO_4_	SH05	5	50	180
	SH25	25		420
0.3 M C_2_H_2_O_4_	SO38	38	50	600
	SO50	50		900
1.1 M H_3_PO_4_	SP60	60	30	130
	SP90	90		300

After anodization, the samples were treated with alumina etchant solution to widen the nano-pores. The etching rate relies on the chemical composition of the porous oxide layer; that again depends on the anodizing electrolyte and its concentration. Several experiments were performed to optimize the pore-widening step for the three electrolyte solutions. Table 3 gives the optimized values of time and temperature for different electrolyte solutions. The sheets were thoroughly rinsed with DI water and ultrasonicated in acetone for five minutes at 27 °C in order to remove any loosely bound chemicals left on the surface. After ultrasonication, the sheets were again rinsed with DI water.

### 2.2. Sensor designing and fabrication

Parallel plate capacitive sensors were developed where the anodized film works as the sensing layer. The aluminium at the base acts as the bottom electrode. A porous gold layer of 3 nm in thickness was deposited on the anodized alumina.

**Fig. 1:**
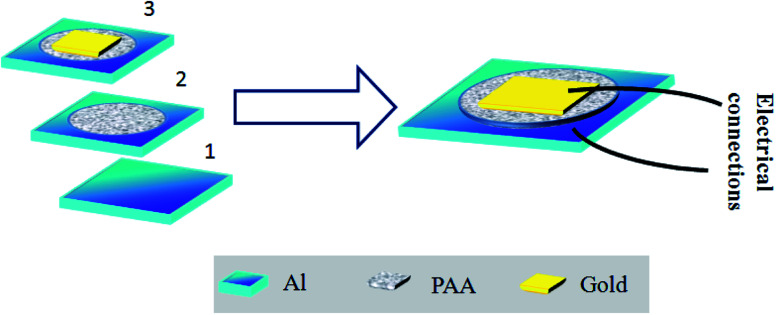
Schematic of the sensor layout; (1): represents the aluminium sheet, which is the starting material and acts as the base electrode of the capacitive sensor. The dimensions of the aluminium sheet are 15 mm × 15 mm. (2): The next step is to anodize the aluminium sheet. Therefore, a circular portion of diameter 10 mm is selected for anodization. This PAA film acts as the sensing layer, and (3): finally, Au is deposited on a portion of the anodized film, which serves the purpose of a top electrode. The dimensions of the Au electrode were 5 mm × 5 mm.

**Fig. 2:**
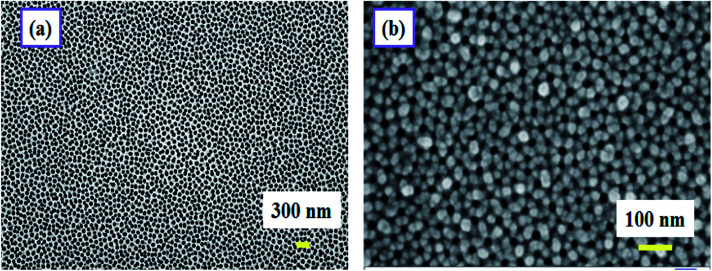
FESEM image of (a) bare and (b) gold-coated PAA.

This gold electrode serves the purpose of the second electrode of a parallel plate capacitive sensor. Fig. 1 schematically describes the formation of the capacitive sensor step by step. Electrical connections were taken out from the top and the bottom electrodes using conductive silver epoxy. The top gold electrode design is deliberately made to cover most of the area of the active PAA layer to serve two purposes: first, it should act as one of the capacitive electrodes, and second, the top Au layer should be porous to allow water vapor to enter through it to be adsorbed on the dielectric, *i.e.* the PAA layer. Therefore, careful optimization of the Au film thickness is very necessary to satisfy both the purposes mentioned. The top gold electrode, deposited *via* a sputtering technique is highly porous and water permeable in nature. We have checked that a gold film thickness of ∼3 nm or slightly thicker proved to be the best for sensing performance. Fig. 2 displays the FESEM images of bare and gold-coated PAA. It can be interpreted from the FESEM image that if the optimized thickness of gold layer is deposited, it will not cover the pores beneath it and thus allows water vapor molecules to permeate through it.

### 2.3. Humidity sensing setup

**Fig. 3:**
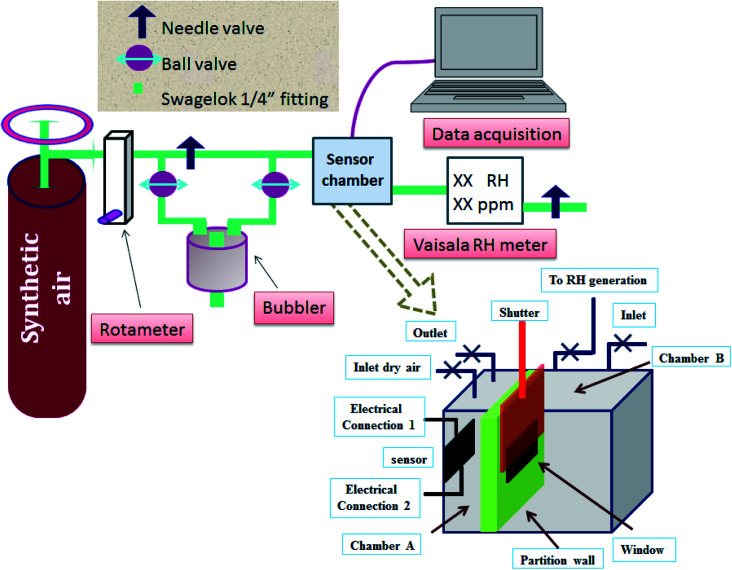
Schematic diagram of the humidity sensing setup.

The humidity sensing characteristics of the as-prepared samples were measured in a home-made humidity sensing setup (Fig. 3), specially designed to make real-time measurements in a saturated humidity environment. The sensor chamber is divided into two unequal parts: part-A and part-B; the volume of part-A is kept to a bare minimum. Humidity of a specific concentration is generated in part-B, and allowed to rise until it attains a pre-set level of humidity concentration to be measured. A commercial sensor is connected to chamber B to monitor the concentration of the saturated humidity level. Once it is achieved, the shutter is opened in a pulsed manner, to allow water vapor flux to directly hit the developed sensor kept in part-A. Dry commercial grade synthetic air (moisture content ∼4 ppm) is used as a carrier gas, and its flow is divided into two paths, as shown in Fig. 3. The dry gas and moist gas (coming *via* the bubbler route) are combined before the gas is allowed to pass through the sensor chamber. Both the chambers, A and B, of the sample chamber are purged separately for 12 h, in order to achieve a stable baseline capacitance, followed by exposure to different humidity concentrations. Sensing chamber A is connected to a data acquisition system (Semiconductor Characterization System, SCS, Keithley 4200), where a test signal of 30 mV (r.m.s) was used to excite the sensor at frequency 5 kHz with no DC bias.

The moist gas then passes through a commercial RH sensor (Vaisala HMT 330). The measurement of sensing parameters, such as the humidity sensing response, response–recovery time, hysteresis and long-term stability, is carried out at room temperature (300 K) and at atmospheric pressure (1.03 bar). The flow rate of the carrier gas is kept fixed at 2 lpm (liter per minute) while performing the experiment.

## 3. Results and discussion

### 3.1. Fabrication parameters of PAA

The anodic polarization has a direct influence on the diameter of the pores.^25^ Depending on the applied voltage, different morphologies with distinctive pore size, interpore spacing, anodic alumina film thickness and electrolyte-driven species, were observed in the PAA nanostructure.^31^ These parameters are easily tunable, and their effectiveness in sensor performance may be checked.

**Fig. 4:**
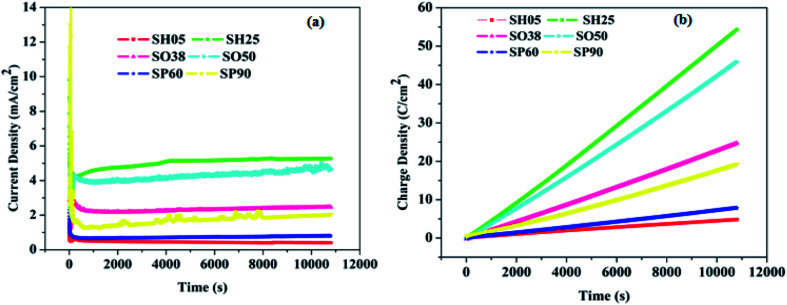
(a) Current density variation with time, and (b) variation of charge density with time during anodization.

Fig. 4(a) shows the variation in current density with time, which is very similar to that of conventional anodizing conducted in potentiostatic mode. It can be observed from Fig. 4(a) that the current density saturates after some time (∼300 seconds). However, the value of the saturation current density (*J*_sat._) is different for each sample. Even for samples anodized in the same electrolyte, the current density is greater for the sample anodized at a higher voltage, than for the sample which is anodized at a low voltage: *e.g. J*_sat.(SH25)_ > *J*_sat.(SH05)_. Similarly, for the sample anodized in oxalic and phosphoric acid: *J*_sat.(SO50)_ > *J*_sat.(SO38)_ and *J*_sat.(SP90)_ > *J*_sat.(SP60)_. Fig. 4(b) shows the variation in charge density with time for the fabricated samples. This varies in accordance with the variation in current density, *e.g.* SH25 > SH05, SO50 > SO38 and SP90 > SP60, and affects the overall water vapor adsorption dynamics. The saturation current density (*J*_sat._) gives an idea of the concentration of incorporated electrolyte anions, *viz.* SO_4_^2−^, C_2_O_4_^2−^ and PO_4_^2−^, which inherently enter into the anodic oxide layer during anodizing. The plots in Fig. 4(a and b) clearly signifies that the concentration of acid-driven anions is greater for the samples having a high value of saturation current density *e.g.* SH25 > SH05, SO50 > SO38 and SP90 > SP60. However, the values for electrolyte-driven anions vary in the ranges: 10–14% in sulfuric acid, 2–3% in oxalic acid, and 6–8% in phosphoric acid solution.^25^

**Fig. 5:**
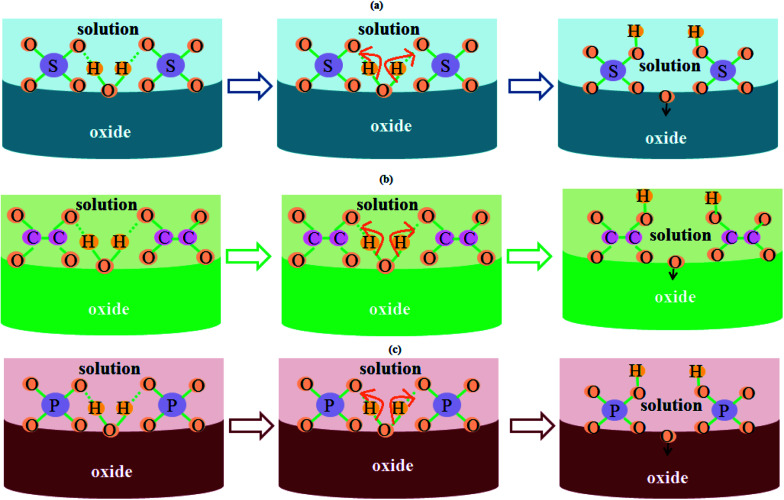
Schematic illustrating the role of electrolyte-driven anions in the growth kinetics of PAA in (a) sulfuric acid solution, (b) oxalic acid solution, and (c) phosphoric acid solution.

Fig. 5 schematically illustrates the role of electrolyte-driven anions in the fabrication process of PAA nanostructures. The H_2_O molecules present in the electrolyte break apart into OH^−^ and O^2−^ anions. The O^2−^ anions assist in the formation of PAA while the OH^−^ anions combine with sulfate anions. Therefore, apart from acid-driven anions, there might be the presence of trapped water molecules producing OH^−^ and O^2−^ anions in the anodic aluminium oxide. The presence of these anions in the anodic oxide structure greatly affects the humidity sensing characteristics, as they influence the water vapor adsorption dynamics.

These anions assist the adsorption of water vapor molecules on the porous oxide structure; hence a significant change in capacitance is observed for the samples with a high concentration of acid-driven anions. This is explained and shown later in Section 3.5 (Fig. 9 and 10) except for samples SH05 and SH25, where the pore diameter dominates over the electrolyte-driven anion concentration to govern the characteristics of the sensor.

**Fig. 6:**
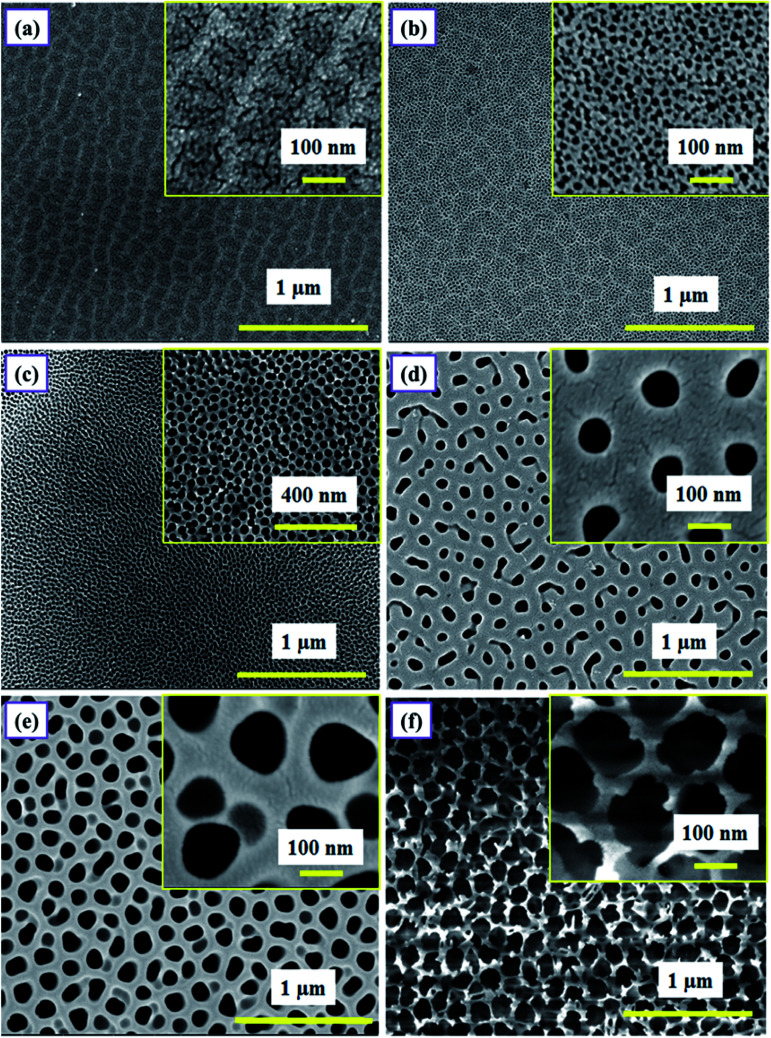
FESEM micrographs of samples (a and b) SH05 and SH25 anodized in sulfuric acid solution, (c and d) SO38 and SO50 anodized in oxalic acid solution, and (e and f) SP60 and SP90 anodized in phosphoric acid solution. The images in the insets display the magnified views of the respective samples.

**Fig. 7:**
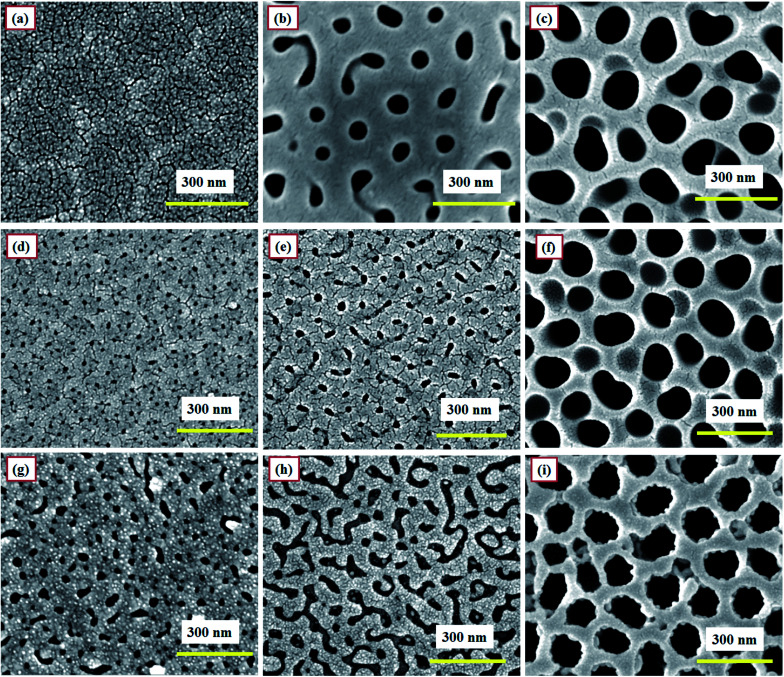
FESEM images of sensors (a) SH05, (b) SO50, and (c) SP60 kept at 50 RH% continuously for 3 months at RT. FESEM images taken 12 months later for bare PAA sections of sensors (d) SH05, (e) SO50, (f) SP60, and gold-coated PAA sections of sensors (g) SH05, (h) SO50, (i) SP60 tested periodically in the humidity range of 3–90 RH%.

**Fig. 8:**
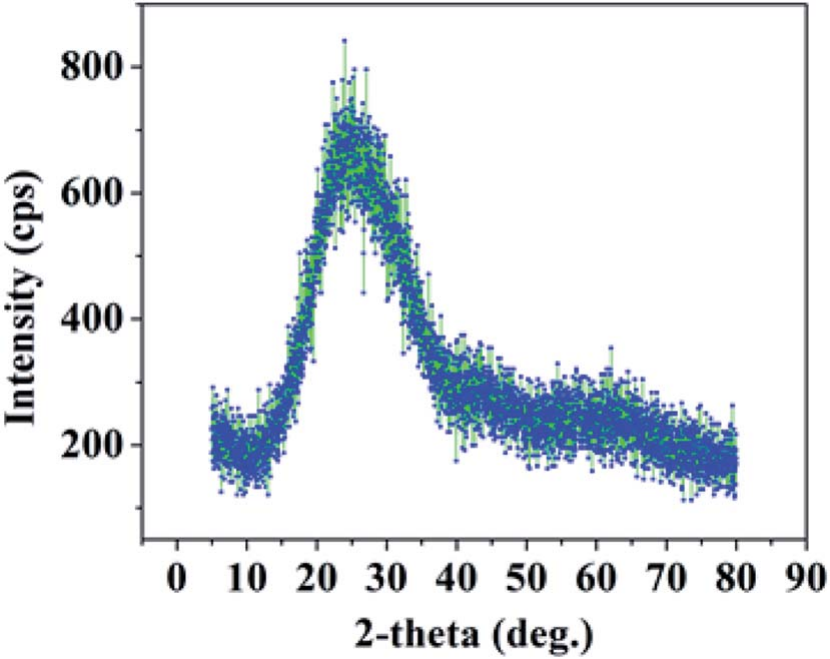
XRD spectrum of PAA.

**Fig. 9:**
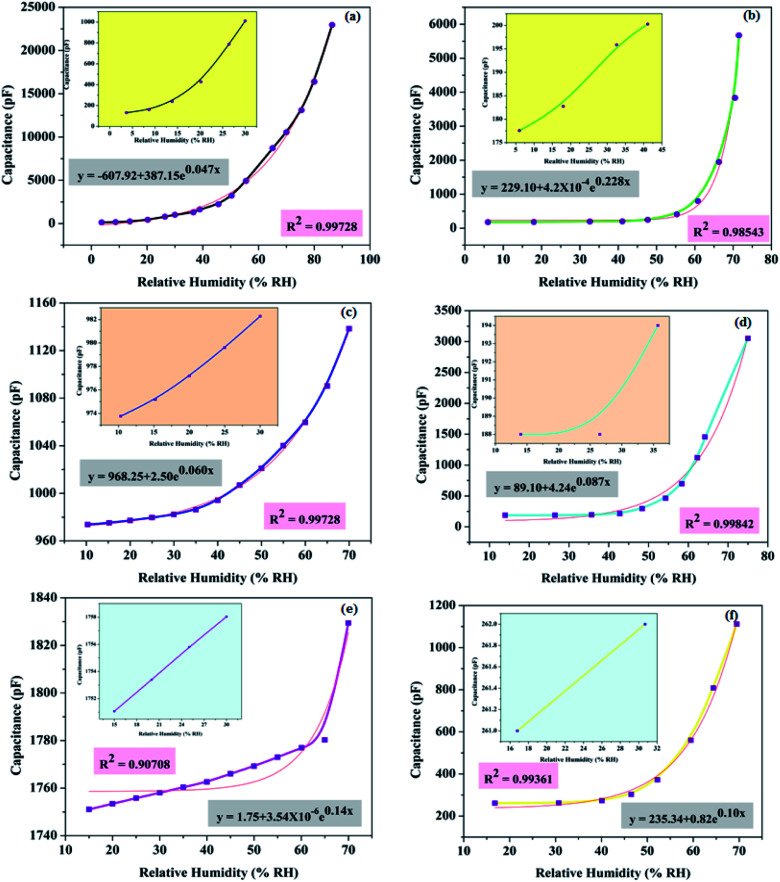
Capacitance responses of the sensors (a) SH05, (b) SH25, (c) SO38, (d) SO50, (e) SP60, (f) SP90 with fitted curves (shown as pink lines). The figures in the insets show the capacitive responses for the low RH range.

**Fig. 10:**
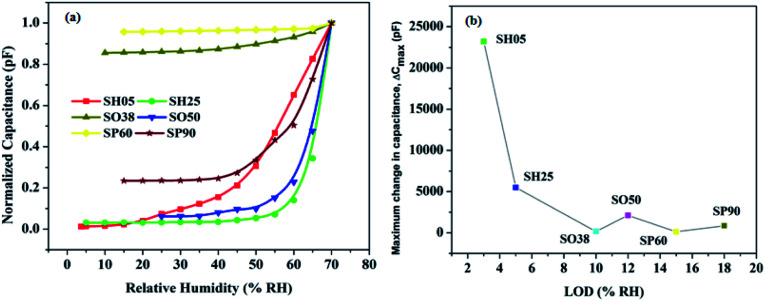
(a) Normalized capacitive response as a function of humidity for the developed sensors, (b) maximum change in capacitance *versus* LOD.

### 3.2. Surface morphology analysis

The surface morphology of as-prepared PAA samples was observed using FESEM (FEI, NOVA NANOSEM 450). The samples were generally coated with a thin layer of gold prior to imaging, to avoid any charging effect caused by the insulating nature of PAA. For imaging, the samples were placed on an aluminum stub with carbon tape and positioned inside the sample chamber. Fig. 6 shows the FESEM images of samples SH05, SH25, SO38, SO50, SP60 and SP90.

The micrographs are displayed at two different scales (Fig. 6); emphasizing the long-range ordering at the micron-scale, and the size of the pores at the nanometer-scale (the images in the insets); in order to precisely analyze the effect of pore size and the overall surface morphology on the humidity sensing response of PAA. The samples anodized in sulfuric acid solution at 5 V and 25 V, *i.e.* SH05 and SH25, are shown in Fig. 6(a and b) with average pore diameters of 5 ± 3 nm and 10 ± 3 nm, respectively. Fig. 6(c, d) and (e, f) show micrographs of samples anodized in oxalic acid (at 38 V and 50 V) and phosphoric acid (at 60 V and 90 V) solution with average pore diameters of 30 ± 5 nm, 74 ± 5 nm and 98 ± 5 nm, 151 ± 5 nm, respectively. The micrographs confirm the formation of self-assembled and uniform pores with long-range ordering.

The pore diameter plays a major role in deciding the sensor characteristics, especially the lower detection limit and sensitivity.^33^ However, the overall surface morphology, *e.g.* the self-assembled long-range ordering and uniformity of the porous structure, also affects the sensing characteristics.^26–29^ It has been observed that, amongst samples with the same pore diameter but differences in long-range ordering and uniformity, a sample with a better pore arrangement gives better results than a sample with an unordered porous structure, in terms of sensitivity and defining the lower detection limit. Therefore, the aforementioned parameters must be optimized for a better humidity sensing response.

### 3.3. Sensor stability and structural integrity analysis

Fig. 7 shows the FESEM images of different sections of moisture-sensitive materials of a developed PAA-based humidity sensor at different times. Two sets of investigations were carried out to check the material stability and integrity. Sensors SH05, SO50 and SP60 were chosen to further check for electrolyte specific surface degradation, if any.

In the first study, after testing, sensors SH05, SO50 and SP60 were kept at a humidity of 50 RH% continuously for 3 months at room temperature (Fig. 7(a)–(c)). It can be observed that the morphologies of the samples remain almost the same and no degradation in the porous structure is observed. Thus it can be concluded that no degradation in morphology is observed after exposing the sensor continuously to medium-range humidity (0–50 RH%).

In the second study, the developed sensors were tested periodically over the complete RH range: *i.e.* 3–90 RH% for 12 months at room temperature. Fig. 7(d)–(f) and (g)–(i) display the FESEM images of bare and gold-coated sections of sensors SH05, SO50 and SP60, respectively. A minor variation in pore size for samples SH05 and SO50 can be observed in the respective FESEM images for bare (Fig. 7(d) and (e)) and gold-coated (Fig. 7(g) and (h)) sections of sensors SH05 and SO50, respectively. However, negligible deterioration was found in their sensing data and this is reflected in Fig. 13, a plot of their aging effect/shelf life.

It can be concluded that upon exposure to high humidity that a minor variation in porous structure is observed for samples anodized in sulfuric and oxalic acid solution, whereas no degradation in structure is observed for samples anodized in phosphoric acid solution. Therefore, a sensor developed from anodization in phosphoric acid solution is found to be particularly useful for applications where continuous monitoring of a high humidity level (>50 RH%) is required. On the other hand, a sensor developed from anodization in sulfuric acid or oxalic acid solution is suitable for monitoring low and medium ranges of humidity (0–50 RH%).

### 3.4 Phase determination of PAA

The phase of PAA fabricated by anodization was determined by carrying out X-ray diffraction measurements (Rigaku, Smartlab) using Cu-Kα radiation, where *λ* = 1.54 Å. Fig. 8 displays the XRD spectrum of PAA, confirming its amorphous nature.^30,31^

Alumina exists in many stable and meta-stable phases; however, only two crystalline phases, *i.e.* α-Al_2_O_3_ and γ-Al_2_O_3_, are important for humidity sensor fabrication. Most of the reported humidity sensors were found to exist in the γ phase of Al_2_O_3_.^32^ The γ-Al_2_O_3_ phase is liable to change to γ-Al_2_O_3_·H_2_O which leads to surface degradation upon exposure to high humidity.^23^ The phase is mainly amorphous in nature with a small crystalline content.^33^ The α-Al_2_O_3_ is expected to be a promising candidate owing to its high thermal stability. Chen in 1991 reported an α-Al_2_O_3_ based humidity sensor fabricated by an anodic spark deposition technique.^34^

### 3.5. Humidity sensor parameter measurement

The humidity sensing performance of PAA samples with different surface morphologies was analyzed by measuring the capacitance of the sensor, as a function of humidity. It can be observed from Fig. 9 that capacitance increases exponentially with increase in humidity. The relationship between sensor capacitance (*y*) and relative humidity (*x*) is represented by the equation

1 *y* = *y*_o_ + *Ae*^*R*_o_*x*^

where the values of *y*_o_, *A*, *R*_o_ are given in the plots (Fig. 9) for each sample. The capacitive response of the as-prepared samples is found to be in excellent agreement with eqn (1) with a value of *R*^2^ ∼ 0.9987; as given separately in each plot (Fig. 9).

The capacitive responses of samples SH05 and SH25 with a small pore diameter, SO38 and SO50 with a medium pore diameter and SP60 and SP90 with a large pore diameter in nano-dimensions, are displayed in Fig. 9(a, b), (c, d) and (e, f), respectively. The insets in Fig. 9 show the capacitive response for the low RH range, where generally the rise in capacitance is almost negligible, as in published reports.^18–24^ Sample SH05 shows an exceptionally high change in capacitance for a low RH range, and the lower detection limit is found to be extended to 3 RH%. We suppose, in the first instance, that these are directly related to its exceptionally small pore diameter. An optimum pore size comparable to the mean free path and size of a water vapor molecule is desirable for adsorption in a porous dielectric material; which is given by the Kelvin equation as2
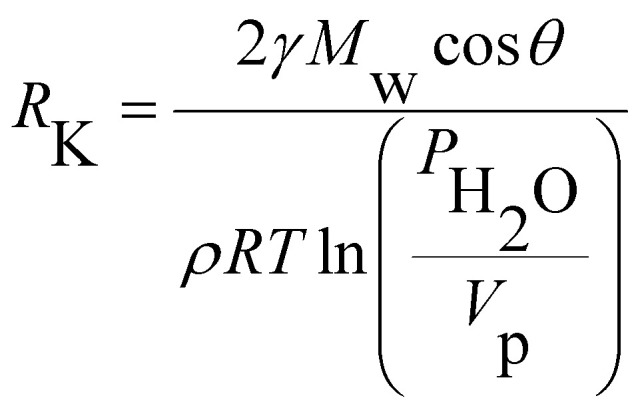
where *R*_K_ is the Kelvin radius, *γ* is the surface tension, *M*_w_ is the molar mass and *ρ* is the density of water vapor molecules. *R* is the universal gas constant, *T* is the temperature, *P*_H_2_O_ is the partial pressure of water and *V*_p_ is the vapor pressure of water.

At a low RH level, especially below 10 RH%, the size of the cluster of water vapor molecules is very small, and, therefore, a very small pore diameter is desirable for moisture detection at a low RH range. Sample SH05, with the smallest pore diameter of 5 nm ± 3 nm among the prepared samples, satisfies the condition and is able to detect at 3 RH% (Fig. 9(a)).

It can be concluded from the plots that, as the pore diameter increases, the maximum change in capacitance decreases and LOD shifts towards a high RH value. This happens because water molecules at low RH level can easily escape due to less scattering with the pore walls or with other molecules, leading to a large mean free path, less loss of kinetic energy and subsequently a high molecular Brownian energy.^35^

Therefore, the higher the pore diameter, the greater the Brownian energy, and the greater is the chance that vapour molecules, at low RH, will not adsorb on the pore walls; and it is evident from Fig. 10 that the minimum detection limit pushes towards a high RH with an increase in pore diameter. Another observation is the effect of anionic concentration on the anodic structure. A sample with a low concentration of incorporated anions in the anodic structure show less change in capacitance; except for samples with exceptionally small pore diameters, *e.g.* SH05 and SH25.

The LOD and sensitivity are dominated by pore diameter. The smaller the diameter of the pores, the lower is the LOD, and the better the sensitivity. However, sensitivity also depends on electrolyte concentration; the larger the concentration of incorporated anions, the larger is the sensitivity. So in order to move towards trace-level detection or detection between trace and RH, an exceptionally small pore diameter along with a higher concentration of electrolyte-driven anions is required.

The comparative normalized capacitive responses of the developed sensors are shown in Fig. 10(a), whereas Fig. 10(b) exhibits the maximum change in capacitance *versus* LOD. Table 4 summarizes the effect of the fabrication dynamics of PAA, *e.g.* electrolyte anion incorporation and pore diameter, on sensor performance.

**Table 4:** Correlation between fabrication parameters and their dynamics on the capacitive humidity sensing response

Electrolyte	Acid-driven anion incorporation (%)	Variation of incorporated anions within the mentioned range^a^	Sample name	Anodization voltage (V)	Pore diameter (nm)	Δ*C*_max._ (total change in capacitance) pF	LOD (% RH)
0.3 M H_2_SO_4_	10–14	↓	SH05	5	5 ± 3	23 209	3
		↑	SH25	25	10 ± 3	5494	5
0.3 M C_2_H_2_O_4_	2–3	↓	SO38	38	30 ± 5	164	10
		↑	SO50	50	74 ± 5	2121	12
1.1 M H_3_PO_4_	6–8	↓	SP60	60	98 ± 5	116	15
		↑	SP90	90	151 ± 5	850	18

a ↓ and ↑ signify low and high concentrations of electrolyte-driven anions specific to the anodizing electrolyte (within the range mentioned in column 2).

**Fig. 11:**
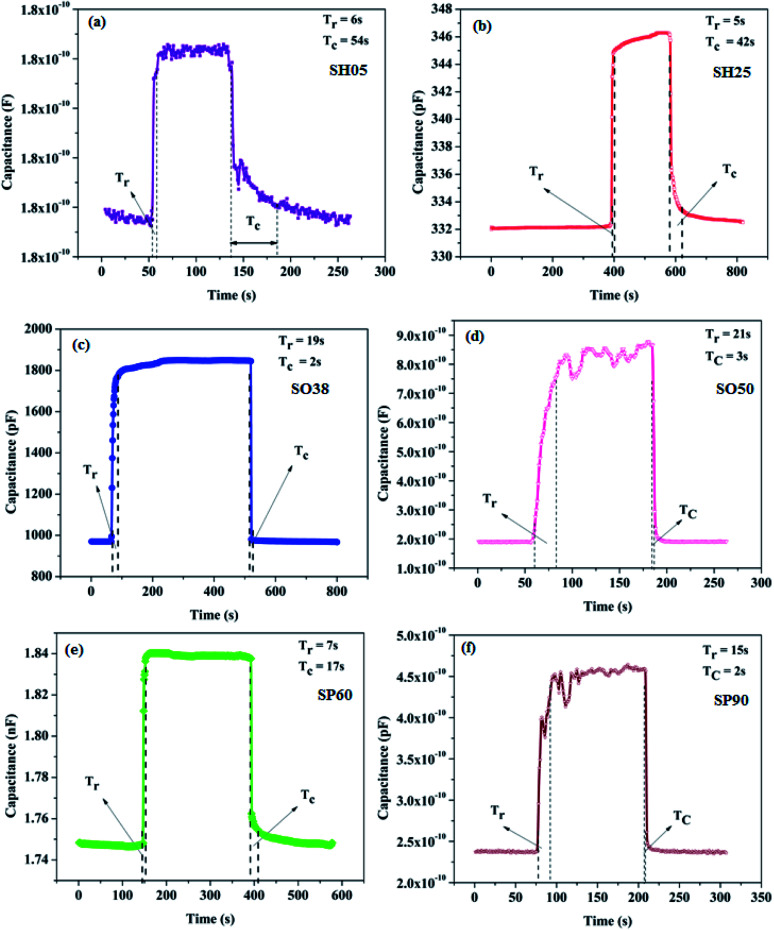
Transient responses exhibiting the response and recovery times of the prepared sensors (a) SH05, (b) SH25, (c) SO38, (d) SO50, (e) SP60 and (f) SP90 at 50 RH%.

In order to determine the response time (*T*_r_) and recovery time (*T*_c_), the sensor is exposed to a step change in humidity: *i.e.* 0–50 RH%. The response time is defined as the time taken to go from 10% to 90% of the maximum change in sensor output; whereas the recovery time is defined as the time taken to recover from 90% to 10% of the maximum change in sensor output. The transient curves exhibiting the response and recovery time of the samples are shown in Fig. 11.

The prepared samples were found to be extremely fast; response and recovery times were found to be within 20 seconds respectively. The recovery times of samples SH05 and SH25 are relatively high, which is attributed to the small pore diameter of the samples, as it takes a longer time for desorption of water molecule from nano-order pores; where water vapor molecules are initially bound chemically to make a chemisorption layer on PAA.^35^

**Fig. 12:**
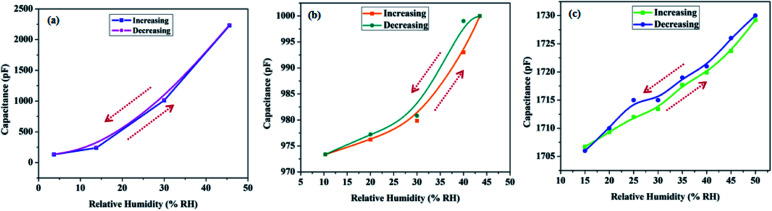
Hysteresis plots of samples (a) SH05, (b) SO38, and (c) SP60.

Hysteresis loss is one such parameter that negates any sensor, however good it is on other fronts. It is measured for all sensors, working on adsorption and desorption phenomena; and is defined as the percentage deviation in capacitance while humidity increases or decreases in a given RH range, *i.e.* 0–50 RH%. It is evident from Fig. 12 that all the prepared samples return to the original capacitance value to a large extent; showing that the sensors are stable with negligible baseline drift. Fig. 12 displays the hysteresis plots of samples SH05, SO38 and SP60, with hysteresis losses of 0.36%, 0.6% and 0.13% at 45% RH, respectively.

**Fig. 13:**
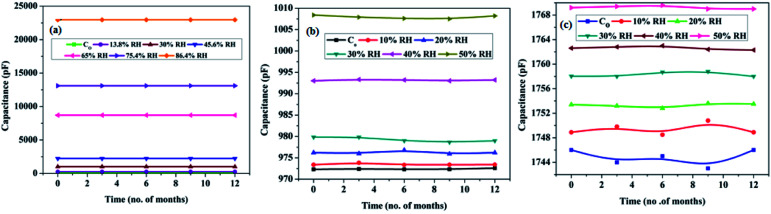
Long-term stability curves of samples (a) SH05, (b) SO38 and (c) SP60.

Drift is an another important sensor parameter, which is useful as far as the long-term utility of the sensor is concerned, and that arises because of an aging effect. The prepared samples were safely stored after testing, and the drift in sensing profile was checked every 3 months for a year. Samples with the smallest pore diameter were found to be the most stable with negligible drift in the sensor output vis-à-vis the others. The long-term stability plots of samples SH05, SO38 and SP60 are shown in Fig. 13.

## Conclusions

We have reported a comprehensive as well as transparent understanding of the humidity sensing mechanism in the developed sensors, taking into account the effect of the diameter and uniformity of self-assembled nano-pores, along with electrolyte-driven anions in the grown PAA nanostructure. The underlying principle of PAA fabrication and its impact on sensor performance were thoroughly investigated; and a mechanism to further reduce the minimum detection limit with improved sensing characteristics was suggested. All the prepared samples exhibit an exponential change in capacitance with an increase in humidity, which is given by the equation *y* = *y*_o_ + *Ae*^*R*_o_*x*^. Sample SH05 with a pore diameter less than 5 nm ± 3 nm, was found to be extremely sensitive and the lower detection limit (LOD) was reduced even further to 3 RH%, an exceptional outcome indeed. Besides, the developed sensor (SH05) was found to be extremely fast with response and recovery times of ∼6 s and ∼54 s, respectively; however, the recovery times of samples SH05 and SH25 were found to be a little longer, which is directly related to the molecular kinetics of vapour molecules in an exceptionally small pore diameter (<11 nm). Sensors prepared *via* anodization in phosphoric acid solution were found to be useful for monitoring high humidity levels (>50 RH%) whereas sensors developed *via* anodization in sulfuric or oxalic acid solution are useful for monitoring low and medium ranges of humidity (0–50 RH%). The long-term drift was also investigated and found to show a negligible shift in capacitance value, ensuring the long-term stability of the developed sensor.

## Conflicts of interest

There are no conflicts to declare.

## Supplementary Material

## References

[cit1] Cho M. Y., Kim S., Kim I. S., Kim E. S., Wang Z. J., Kim N. Y., Oh J. M. (2019). Perovskite-Induced Ultrasensitive and Highly Stable Humidity Sensor Systems Prepared by Aerosol Deposition at Room Temperature. Adv. Funct. Mater..

[cit2] Suematsu K., Sasaki M., Ma N., Yuasa M., Shimanoe K. (2016). Antimony-doped tin dioxide gas sensors exhibiting high stability in the sensitivity to humidity changes. ACS Sens..

[cit3] He P., Brent J. R., Ding H., Yang J., Lewis D. J., O'Brien P., Derby B. (2018). Fully printed high performance humidity sensors based on two-dimensional materials. Nanoscale.

[cit4] Sarkar B., Satapathy D. K., Jaiswal M. (2019). Nanostructuring mechanical cracks in a flexible conducting polymer thin film for ultra-sensitive vapor sensing. Nanoscale.

[cit5] Li Y., Zhang L., Yang M., He W. (2019). Improving humidity sensing properties of copolymer-based polyelectrolytes by modifying the chemical structure and content of the comonomers. Sens. Actuators, B.

[cit6] Li B., Tian Q., Su H., Wang X., Wang T., Zhang D. (2019). High sensitivity portable capacitive humidity sensor based on In2O3 nanocubes-decorated GO nanosheets and its wearable application in respiration detection. Sens. Actuators, B.

[cit7] Zhang Y., Duan Z., Zou H., Ma M. (2018). Drawn a facile sensor: A fast response humidity sensor based on pencil-trace. Sens. Actuators, B.

[cit8] Jiang K., Zhao H., Dai J., Kuang D., Fei T., Zhang T. (2016). Excellent humidity sensor based on LiCl loaded hierarchically porous polymeric microspheres. ACS Appl. Mater. Interfaces.

[cit9] Sharma K., Islam S. S. (2016). Optimization of porous anodic alumina nanostructure for ultra high sensitive humidity sensor. Sens. Actuators, B.

[cit10] Kumar S., Islam T., Raina K. K. (2018). Study of long term drift of aluminum oxide thin film capacitive moisture sensor. IEEE Trans. Device Mater. Reliab..

[cit11] Guo H., Lan C., Zhou Z., Sun P., Wei D., Li C. (2017). Transparent, flexible, and stretchable WS 2 based humidity sensors for electronic skin. Nanoscale.

[cit12] Yu Y., Zhang Y., Jin L., Chen Z., Li Y., Li Q., Yao J. (2018). A Fast Response−Recovery 3D Graphene Foam Humidity Sensor for User Interaction. Sensors.

[cit13] Trung T. Q., Ramasundaram S., Lee N. E. (2017). Transparent, stretchable, and rapid-response humidity sensor for body-attachable wearable electronics. Nano Res..

[cit14] http://hmagrp.com/wp-content/uploads/2018/08/INS-DS-0147-M-Series-Moisture-Probes.pdf

[cit15] https://cosaxentaur.com/resources/files/1036/ProcMoistAnEssSCVP_BrXEn.pdf

[cit16] https://cosaxentaur.com/resources/files/1031/Hygrocontrol_-_Type_81-82-RH-10-2015.pdf

[cit17] https://www.shawmeters.com/wp-content/uploads/2018/09/SADP-Portable-Dewpoint-Meter.pdf

[cit18] Chen S. W., Khor O. K., Liao M. W., Chung C. K. (2014). Sensitivity evolution and enhancement mechanism of porous anodic aluminum oxide humidity sensor using magnetic field. Sens. Actuators, B.

[cit19] Balde M., Vena A., Sorli B. (2015). Fabrication of porous anodic aluminium oxide layers on paper for humidity sensors. Sens. Actuators, B.

[cit20] Khanna V. K., Nahar R. K. (1984). Effect
of moisture on the dielectric properties of porous alumina films. Sens. Actuators.

[cit21] Jin Z., Meng F., Liu J., Li M., Kong L., Liu J. (2011). A novel porous anodic alumina based capacitive sensor towards trace detection of PCBs. Sens. Actuators, B.

[cit22] Kim Y., Jung B., Lee H., Kim H., Lee K., Park H. (2009). Capacitive humidity sensor design based on anodic aluminum oxide. Sens. Actuators, B.

[cit23] Yao L., Zheng M., Li H., Ma L., Shen W. (2009). High-performance humidity sensors based on high-field anodized porous alumina films. Nanotechnology.

[cit24] Nahar R. K. (2000). Study of the performance degradation of thin film aluminum oxide sensor at high humidity. Sens. Actuators, B.

[cit25] Sulka G. D. (2008). Highly ordered anodic porous alumina formation by self-organized anodizing. Nanostruct. Mater. Electrochem..

[cit26] Pandey M., Sharma K., Islam S. S. (2019). Wide Range RH Detection with Digital Readout: Niche Superiority in Terms of Its Exceptional Performance and Inexpensive Technology. Adv. Mater. Phys. Chem..

[cit27] Pandey M., Mishra P., Saha D., Sengupta K., Islam S. S. (2014). Development of commercial trace moisture sensor: a detailed comparative study on microstructural and impedance measurements of two phases of alumina. Electron. Mater. Lett..

[cit28] Pandey M., Tyagi K., Mishra P., Saha D., Sengupta K., Islam S. S. (2012). Nanoporous morphology of alumina films prepared by sol–gel dip coating method on alumina substrate. J. Sol-Gel Sci. Technol..

[cit29] Pandey M., Mishra P., Saha D., Islam S. S. (2013). Polymer optimization for the development of low-cost moisture sensor based on nanoporous alumina thin film. J. Adv. Ceram..

[cit30] Gangwar J., Gupta B. K., Tripathi S. K., Srivastava A. K. (2015). Phase dependent thermal and spectroscopic responses of Al_2_O_3_ nanostructures with different morphogenesis. Nanoscale.

[cit31] Lee W., Park S. J. (2014). Porous anodic aluminum oxide: anodization and templated synthesis of functional nanostructures. Chem. Rev..

[cit32] Chen Z., Lu C. (2005). Humidity sensors: a review of materials and mechanisms. Sens. Lett..

[cit33] Islam T., Nimal A. T., Mittal U., Sharma M. U. (2015). A micro interdigitated thin film metal oxide capacitive sensor for measuring moisture in the range of 175–625 ppm. Sens. Actuators, B.

[cit34] Chen Z., Jin M. C., Zhen C., Chen G. H. (1991). Properties of Modified Anodic-Spark-Deposited Alumina Porous Ceramic Films as Humidity Sensors. J. Am. Ceram. Soc..

[cit35] Sharma K., Alam N., Islam S. S. (2020). New concept in Humidity sensing: Role of Molecular Brownian energy and probabilistic Mean free path to Differentiate RH- and Trace level Detection. ACS Appl. Mater. Interfaces.

